# Identification and Expression Analysis of Chalcone Synthase Gene Family in Tartary Buckwheat

**DOI:** 10.3390/genes16040451

**Published:** 2025-04-14

**Authors:** Qinghai Wang, Yanhua Jia, Xin Lin, Lu Tan, Hanmei Du, Anhu Wang

**Affiliations:** 1 Panxi Crops Research and Utilization Key Laboratory of Sichuan Province, Xichang University, Xichang 615013, China; tanlu19910222@163.com (L.T.); duhanmei1027@163.com (H.D.); 13795660264@163.com (A.W.); 2 School of Resources and Environment, Xichang University, Xichang 615013, China; jyh735680@163.com; 3 School of Agricultural Sciences, Xichang University, Xichang 615013, China; 18990341239@163.com

**Keywords:** Tartary buckwheat, chalcone synthase, gene family, genome−wide identification, expression analysis

## Abstract

Background: Chalcone synthase (CHS) functions as a pivotal and initiating enzyme in the flavonoid biosynthesis pathway within plants, playing a crucial role in the accumulation and metabolic processes of flavonoids. Despite its importance, there has been no comprehensive analysis or detailed description of the *CHS* gene family members specifically in Tartary buckwheat. Methods: Based on a comprehensive analysis using multiple bioinformatics approaches and quantitative real−time PCR (qRT−PCR) technology, this study systematically identified and characterized the *CHS* gene family members from the complete genome sequence of Tartary buckwheat. Results: In this study, we identified a total of 14 *FtCHS* genes (*FtCHS1*−*FtCHS14*) in Tartary buckwheat. Analysis of gene structure and protein motifs showed that most *FtCHS* genes consist of two exons and a single intron, featuring conserved Chal−sti−synt_N and Chal−sti−synt_C domains. Phylogenetic studies suggested that *FtCHS* genes can be categorized into four primary groups: Groups I, II, III, and IV. Further analysis of the promoter regions revealed that the *FtCHS* family genes contain multiple cis−acting elements that respond to light, plant hormones, stress, and developmental cues. By combining phylogenetic analysis with gene expression data, we found that the genes in Group II (*FtCHS3*, *FtCHS4*, *FtCHS5*, and *FtCHS6*) exhibit significantly elevated expression levels specifically in flowers. Conclusions: Our study indicated that *FtCHS* is a gene superfamily comprising at least four functional members. The expression patterns of these *FtCHS* genes suggest their probable involvement in flower−related biological processes in Tartary buckwheat. This work provides fundamental insights into the comprehensive understanding of the functional roles of the *CHS* gene family in Tartary buckwheat.

## 1. Introduction

Flavonoids, a diverse class of important secondary metabolites, are ubiquitously present in vegetables, fruits, cereals, herbs, tea, and other plants [1,2,3]. As of now, over 10,000 flavonoid compounds have been extracted and chemically identified [2]. These compounds fulfill essential functions in various aspects of plant physiology, including seed development, fruit ripening, flower pigmentation, pollination, auxin transport, and as signaling molecules in plant–microorganism interactions [4]. Plants also employ flavonoids to alleviate both biotic and abiotic stresses [5]. For instance, specific flavonoids function as pigments in fruits and flowers, which not only attract pollinators but also offer protection against herbivores, bacterial and fungal pathogens, and environmental stressors such as UV radiation [5]. The synthesis of these compounds takes place at the convergence of the shikimate and acetate pathways. The shikimate pathway provides precursors for coumarin CoA synthesis, while the acetate pathway supplies malonyl−CoA units that are critical for C2 elongation catalyzed by chalcone synthase [6].

**Figure 1:**
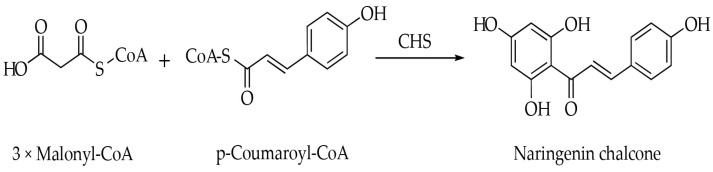
Reaction scheme shows CHS catalyzing the condensation of three malonyl−CoA molecules with *p*−coumaroyl−CoA to produce naringenin chalcone.

Chalcone synthase (CHS, E.C. 2.3.1.74), a key enzyme within the type III polyketide synthase (PKS) superfamily, is crucial for the flavonoid biosynthesis pathway [7]. This enzyme facilitates the condensation of one molecule of *p*−coumaroyl-CoA with three molecules of malonyl−CoA, resulting in the formation of naringenin chalcone (Figure 1). Naringenin chalcone acts as a precursor for a variety of flavonoid compounds [6]. Thus far, numerous *CHS* genes have been identified across different plant species [8,9,10,11]. These genes generally show high nucleotide similarities and encode proteins with molecular weights between 20 and 62 kDa [8,9,10,11]. The encoded proteins feature a catalytic triad of Cys−His−Asn (CHN) at their active sites [12]. Additionally, *CHS* genes exhibit diverse expression patterns and are characterized by specific tissue distribution. For example, in *Cassia alata*, three *CHS* genes (*CalCHS1*, *CalCHS2*, and *CalCHS3*) are predominantly expressed in young roots [13]. In *Pisum sativum*, *PsCHS1*, *PsCHS2* and *PsCHS8* are expressed in both roots and flowers, while *PsCHS3*, *PsCHS4* and *PsCHS5* exhibit root−specific expression [14]. Given their critical roles in multiple biological processes, the regulatory and functional aspects of *CHS* genes have been extensively studied in various plant species, including *Arabidopsis thaliana* [15], *Oryza sativa* [16], *Zea mays* [17], *Gossypium barbadense* [8], *Solanum lycopersicum* [18], *Salvia miltiorrhiza* [19], *Rhododendron delavayi* [20], and *Zostera marina* [21]. Therefore, a comprehensive analysis of the *CHS* gene family is necessary for further elucidating its function and regulatory mechanisms in plant flavonoid biosynthesis.

Tartary buckwheat (*Fagopyrum tataricum*), belonging to the eudicot family Polygonaceae, is a significant minor cereal crop noted for its rich flavonoid content, especially rutin and quercetin, both of which are renowned for their health−promoting properties [22]. To date, multiple genes associated with flavonoid biosynthesis have been identified and functionally characterized in this crop. These include the UDP−glycosyltransferase gene *FtUGT79A15* [23] and transcription factor genes such as *FtMYB3* [24], *FtMYB6* [25], *FtMYB8* [26], *FtMYB102* [27], *FtbHLH4* [27], *FtbZIP85* [28], *FtBPM3* [29,30], *FtERF-EAR3* [30], and *FtTT8* [31]. Previous transcriptome studies have identified 11 putative homologous *CHS* genes in Tartary buckwheat [32], of which only three have been successfully cloned [33]. The completion of the whole genome assembly of Tartary buckwheat in 2017 has facilitated comprehensive investigations into various flavonoid synthesis−related gene families [34]. Hence, conducting a genome−wide identification and comprehensive analysis of the *CHS* gene family in Tartary buckwheat is valuable for gaining deeper insights into its essential characteristics and roles within the flavonoid synthesis pathway.

In this study, we discovered a total of 14 *CHS* genes within the Tartary buckwheat genome. We performed a comprehensive analysis of these *CHS* gene family members, examining various aspects such as their protein physicochemical characteristics, chromosomal positions, gene duplication occurrences, phylogenetic relationships, gene structures, conserved motifs, protein sequence features, and promoter elements. Furthermore, we investigated their tissue−specific expression patterns using qRT−PCR. This work offers a crucial foundation for understanding the roles of *CHS* gene family members in regulating flavonoid biosynthesis in Tartary buckwheat. Also, it may provide promising candidate genes for accelerating the development of high-flavonoid varieties through molecular breeding in future research.

## 2. Materials and Methods

### 2.1. Plant Materials and Sample Collection

The experiment utilized Tartary buckwheat (cv. Xiqiao 2) as the plant material, which was cultivated in flower pots under natural conditions with ambient temperatures ranging from approximately 18 to 30 °C. After eight weeks, different plant tissues, including roots, stems, leaves, flowers, and seeds, were harvested for subsequent analysis. All samples were immediately submerged in the RNAsafer II reagent (Omega Bio−Tek, Norcross, GA, USA) and rapidly frozen in liquid nitrogen for 30 s. They were subsequently stored at −80 °C until RNA extraction was conducted. To ensure RNA integrity and reliable gene expression results, the entire workflow, from sampling to RNA extraction, should be completed within two weeks.

### 2.2. Genome−Wide Identification of the CHS Family Members in Tartary Buckwheat

Firstly, we retrieved the complete genome data, encompassing genome sequences, protein sequences, and genome annotation files from the Tartary buckwheat genome database, MBKbase (https://www.mbkbase.org/Pinku1/ (accessed on 8 January 2025)) [34]. To identify CHS proteins in Tartary buckwheat, we conducted BLASTP searches against the Tartary buckwheat protein dataset using previously reported CHS protein sequences from *O. sativa* [16], *G. barbadense* [8], *Chrysanthemum nankingense* [9], and *Vaccinium corymbosum* [11] as queries, with an e−value threshold of less than 1.0 × 10^−10^. The putative CHS proteins were validated by screening for Hidden Markov Model (HMM) domain profiles (PF00195 and PF02797) using HMMER 3.2.1 software [35]. To further validate the candidate FtCHS proteins, we cross−referenced them using the InterPro database (http://www.ebi.ac.uk/interpro/ (accessed on 10 January 2025)) [36] and the NCBI−CDD search tool (https://www.ncbi.nlm.nih.gov/Structure/cdd/wrpsb.cgi (accessed on 10 January 2025)) [37]. This process confirmed the presence of both the Chal−sti−synt_N domain (PF00195) and the Chal−sti−synt_C domain (PF02797). Only those sequences that included these two domains were considered as members of the *FtCHS* gene family for further analysis. Moreover, we utilized the ExPASy website (https://web.expasy.org/protparam/ (accessed on 14 January 2025)) [38] to determine the fundamental physicochemical properties of each FtCHS protein. Additionally, the subcellular localization of these proteins was predicted using the Cell−PLoc online tool (http://www.csbio.sjtu.edu.cn/bioinf/Cell-PLoc/ (accessed on 14 January 2025)) [39].

### 2.3. Chromosomal Location and Gene Duplication Analysis of FtCHS Genes

The analysis presented in this section was conducted using TBtools−II software [40]. The chromosomal position information for the *FtCHS* genes was derived from the genome annotation file of Tartary buckwheat. To visualize chromosomal localization, we employed the “Gene Location Visualize from GTF/GFF” tool within TBtools−II [40]. Gene duplication analysis was performed using the “Quick Run MCScanX” tool, and the Ka and Ks values for duplicated genes were calculated using the “Simple Ka/Ks Calculator” tool.

### 2.4. Phylogenetic Analysis of FtCHS Genes

The phylogenetic analysis of FtCHS proteins was constructed using MEGA 11 software [41]. Homologous CHS proteins from various plant species were obtained from GenBank (Table S1). The protein sequences were aligned utilizing the MUSCLE algorithm, and an unrooted phylogenetic tree was constructed employing the neighbor−joining (NJ) method. The tree was based on a bootstrap analysis of 1500 replicates under the JTT amino acid substitution model. Visualization and editing of the final phylogenetic tree were performed using FigTree v1.4.4 software (http://tree.bio.ed.ac.uk/software/figtree/ (accessed on 15 January 2025)).

### 2.5. Gene Structures, Conserved Motifs, and Sequence Features of FtCHS Genes

The exon−intron architecture of the *FtCHS* genes was examined utilizing the “Visualize Gene Structure” tool in TBtools−II [40]. To detect conserved motifs within each FtCHS protein, we employed the MEME Suite (https://meme-suite.org/meme/ (accessed on 16 January 2025)) [42] with the following settings: a maximum of 20 motifs, motif width ranging from 6 to 200 amino acids (aa), and an e−value threshold of less than 1.0 × 10^−10^. The detected conserved motifs were subsequently visualized using the “Visualize MEME/MAST Motif Pattern” function in TBtools−II [40].

Drawing from the crystal structure analysis of MsCHS2 protein from *Medicago sativa* [43], we adopted its secondary structure as a reference model to investigate the secondary structures of the FtCHS proteins. This investigation was carried out using two web−based platforms: RCSB PDB (https://www.rcsb.org/ (accessed on 16 January 2025)) [44] and ESPript 3.0 (https://espript.ibcp.fr/ESPript/cgi-bin/ESPript.cgi (accessed on 16 January 2025)) [45]. Furthermore, a multiple sequence alignment involving MsCHS2, FtCHSs, and additional plant CHS proteins was executed using the MUSCLE program within MEGA 11 [41].

### 2.6. Promoter Element Analysis of FtCHS Genes

For promoter analysis, the 2000 base pair (bp) region upstream of the start codon (ATG) for all *FtCHS* genes was extracted. To identify *cis*−acting elements, the online tool PlantCARE (http://bioinformatics.psb.ugent.be/webtools/plantcare/html/ (accessed on 20 January 2025)) [46] was employed. Following this, the identified elements were counted and subjected to further analysis using Excel 2019.

### 2.7. Tissue Expression Analysis of FtCHS Genes

To isolate total RNA from the plant samples, the E.Z.N.A. ^®^ Plant RNA Kit supplied by Omega Bio−Tek (Norcross, GA, USA) was utilized. Following this, 1 μg of the extracted total RNA from every sample underwent reverse transcription into single−stranded cDNA templates using the PrimeScriptTM RT reagent Kit with gDNA Eraser from TaKaRa (Dalian, China), adhering to the protocol provided by the manufacturer. The design of gene−specific primers for qRT−PCR was accomplished through Primer Premier 5.0 software (Table S2). For normalization purposes, *Histone 3* (*H3*, HM628903.1) functioned as the reference gene. Each PCR reaction mixture was prepared to a final volume of 25 µL, including 12.5 µL of TB Green^®^ Premix Ex Taq II (2X) (TaKaRa, Dalian, China), 1.0 µL forward primer at a concentration of 10 µM, 1.0 µL reverse primer at 10 µM, 2.0 µL of cDNA template (with a total amount not exceeding 100 ng), and 8.5 µL of sterile distilled water. The amplification conditions for qRT−PCR involved an initial denaturation step at 95 °C for 30 s, succeeded by 40 cycles of denaturation at 95 °C for 5 s and annealing/extension at 60 °C for 30 s. Each sample was subjected to three independent biological replicates, and the relative expression levels of genes were calculated using the 2^−∆∆Ct^ method [47].

## 3. Results

### 3.1. Identification of the CHS Gene Family Members in Tartary Buckwheat

Through protein homology alignment and conserved domain identification, a total of 14 *CHS* gene family members were identified in the Tartary buckwheat genome. These genes were designated as *FtCHS1* to *FtCHS14* based on their chromosomal positions (Table 1). The open reading frames (ORFs) of the *FtCHS* genes ranged from 1035 to 1188 bp, with corresponding coding sequences (CDSs) ranging from 1032 to 1185 bp. The physicochemical characteristics of the FtCHS proteins are outlined in Table 2. The length of FtCHS proteins varied from 344 to 395 aa, corresponding to molecular weights (*M*w) between 37,702.24 and 43,728.64 Da. The isoelectric points (pI) ranged from 5.38 to 7.58, with the majority (13/14) being below 7.0 (except for FtCHS6), indicating that these proteins were predominantly acidic. Instability index analysis revealed that most (11/14) were stable proteins, with an instability index below 40.0 (except for FtCHS2, FtCHS11, and FtCHS12). Grand average of hydropathicity (GRAVY) analysis indicated that, except for FtCHS7, the other 13 FtCHS proteins exhibited hydrophilic properties (GRAVY values < 0). Subcellular localization analysis predicted that all FtCHS proteins were located within the cytoplasm.

### 3.2. Chromosomal Location and Gene Duplication

**Figure 2:**
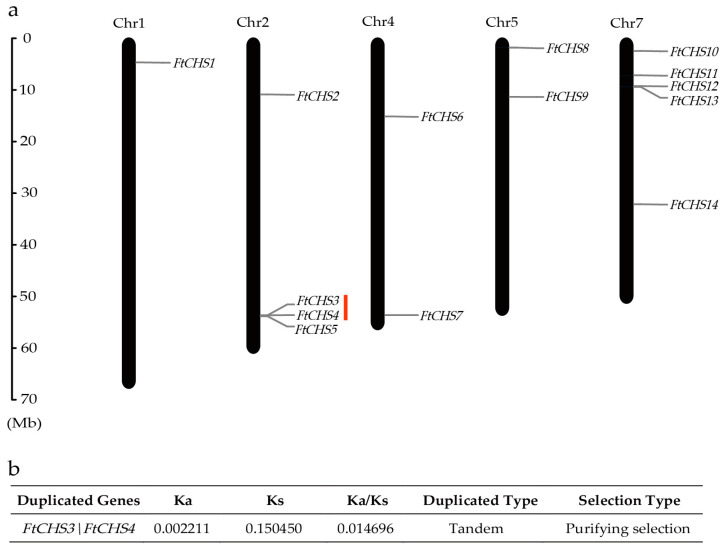
Analysis of chromosome location and gene duplication for *FtCHS* genes. (**a**) Distribution of *FtCHS* genes across chromosomes. The “Chr” label above each bar denotes the chromosome number in Tartary buckwheat. (**b**) Analysis of the Ka/Ks ratio for duplicated *FtCHS* genes. Tandemly repeated genes are indicated with a red line.

Chromosome localization analysis demonstrated that the 14 *FtCHS* genes were unevenly distributed across five chromosomes: Chr1, Chr2, Chr4, Chr5, and Chr7 (Figure 2a). Chr7 contained the highest number of *FtCHS* genes (five), followed by Chr2 with four genes. Chr4 and Chr5 each had two genes, while Chr1 harbored only one. Gene family expansion can result from gene duplication events such as tandem and segmental duplications, which are crucial for driving gene family diversification during evolution [48]. In our investigation of duplication events within the *FtCHS* gene family, we identified a tandem duplication involving the gene pair *FtCHS3* and *FtCHS4* on Chr2 (Figure 2a). Notably, no segmental duplications were detected in this gene family. Furthermore, the Ka/Ks ratio for the duplicated gene pair *FtCHS3* and *FtCHS4* was less than 1.0 (Figure 2b), indicating purifying selection and suggesting a high level of conservation within this gene family.

### 3.3. Phylogenetic Analysis and Classification of FtCHS Genes

**Figure 3:**
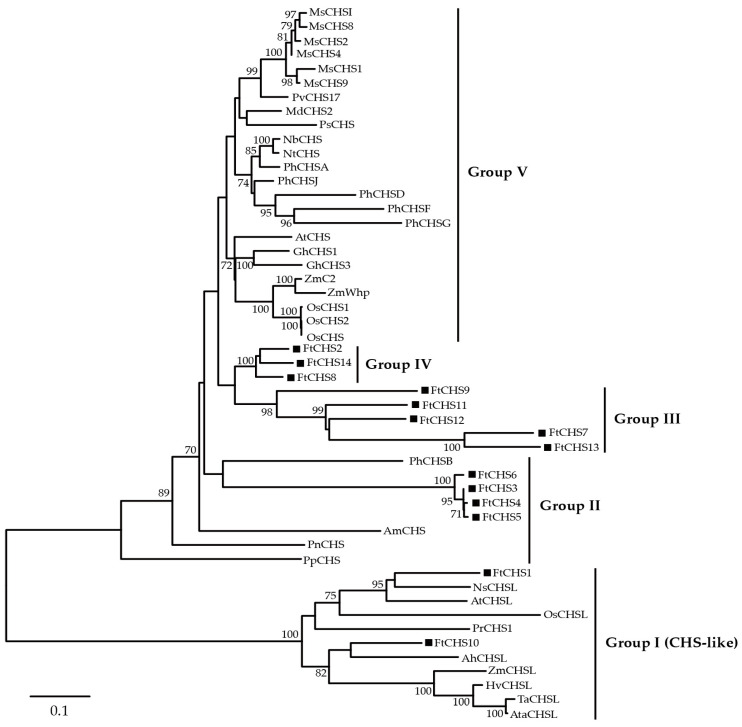
Molecular phylogenetic analysis of chalcone synthases (CHSs) from multiple plant species. CHS proteins from additional plants are detailed in Table S2. FtCHS proteins are highlighted with black squares. Bootstrap values ≥ 70% are indicated.

To investigate the phylogenetic relationships of *CHS* genes in plants, a neighbor−joining (NJ) approach was utilized to construct a phylogenetic tree using 51 protein sequences derived from various plant species (Figure 3). The resulting analysis categorized plant CHS proteins into five distinct groups: Groups I, II, III, IV, and V. Remarkably, 14 FtCHS proteins were distributed among four of these groups (I−IV). Group I consisted of FtCHS1 and FtCHS10, which grouped closely with characterized CHS−like proteins from other plants, such as *A. thaliana* AtCHSL and *Nicotiana sylvestris* NsCHSL. Notably, NsCHSL has been confirmed as an anther−specific CHS−like (ASCL) enzyme [49], suggesting that FtCHS1 and FtCHS10 may also possess ASCL functionality. Group II consisted of FtCHS3, FtCHS4, FtCHS5, and FtCHS6, which clustered with CHS proteins from *Petunia hybrida* (PhCHSB), *Antirrhinum majus* (AmCHS), *Psilotum nudum* (PnCHS), and *Physcomitrella patens* (PpCHS). PpCHS is recognized as one of the earliest identified CHS enzymes [50]. In contrast, the remaining eight FtCHS proteins in Groups III and IV did not cluster with any homologous CHS proteins from other plants, indicating a more distant evolutionary relationship relative to other plant CHS proteins.

### 3.4. Gene Structures of FtCHS Genes

**Figure 4:**
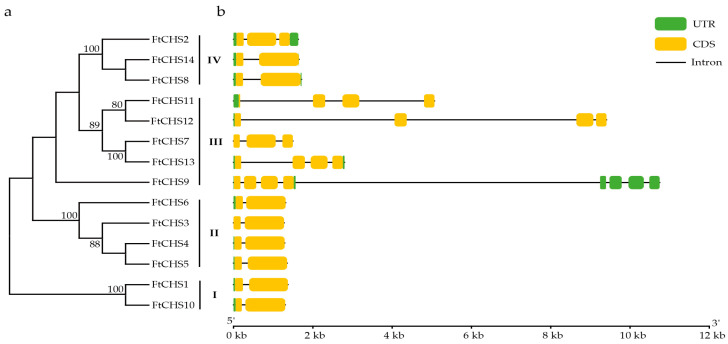
Phylogenetic analysis and gene structures of *FtCHS* genes. (**a**) Phylogenetic analysis of *FtCHS* genes, showing bootstrap support values of ≥70%. (**b**) Exon and intron organization of *FtCHS* genes.

The exon−intron organization of *FtCHS* genes was determined by aligning the mRNA sequences with their respective genomic sequences. The number of exons in the *FtCHS* gene family varied from two to eight. Notably, members belonging to the same subfamily displayed highly similar exon−intron structures (Figure 4). Groups I and II consistently displayed a two−exon structure, while Group IV contained either two or three exons. Genes in Group III generally possessed a greater number of exons and longer introns compared to other groups. For instance, the *FtCHS9* gene, spanning over 10 kb and comprising 8 exons and 7 introns, was identified as the longest gene in the *FtCHS* gene family. Its first four exons encoded the coding sequence (CDS), while the last four exons entirely constituted the 3’ untranslated region (UTR). Overall, the analysis of the gene structure showed that most members (8/14) of the *CHS* gene family in Tartary buckwheat shared a common gene architecture, featuring two exons and a single intron.

### 3.5. Conserved Motifs and Sequence Features of FtCHS Proteins

**Figure 5:**
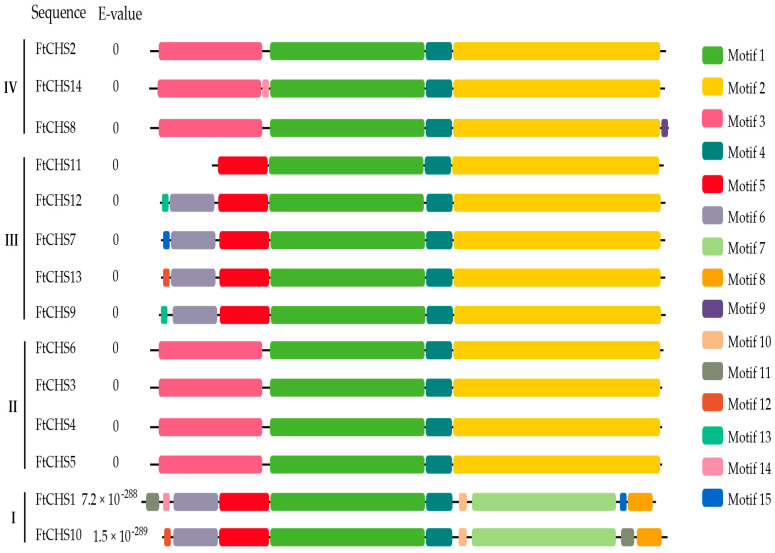
Conserved motif analysis of FtCHS proteins. Various conserved motifs are indicated by distinct colors. The labels I, II, III, and IV on the left side of the image correspond to FtCHS proteins in phylogenetic groups I, II, III, and IV, respectively.

**Figure 6:**
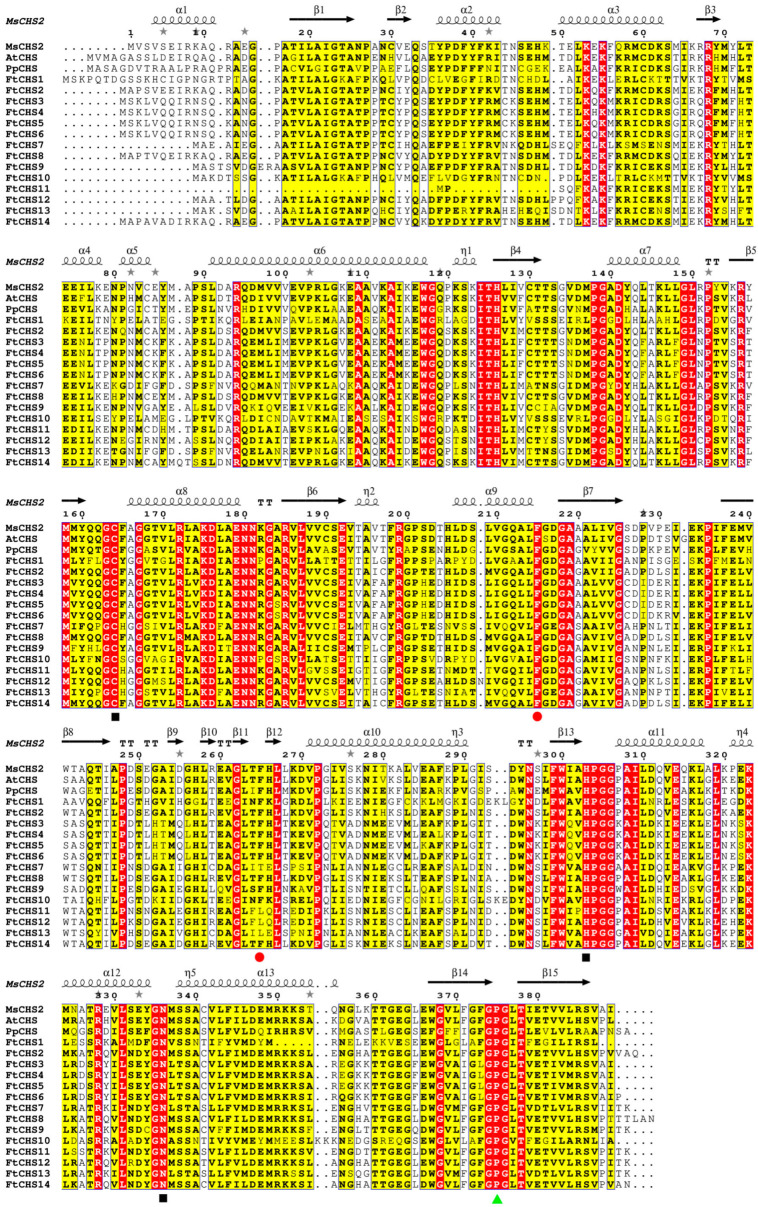
Multiple sequence alignment of FtCHS proteins in comparison with CHSs from other plants. The secondary structure of MsCHS2 is depicted in the top line, where red segments highlight strictly conserved sequences. α−helices are indicated by black wavy lines, while β−sheets are denoted by black arrows. At the bottom, black squares highlight the Cys−His−Asn (CHN) catalytic triad, while red dots indicate CoA−binding residues, and green triangles emphasize the CHS family−specific Pro375 residue. Protein sequences for *M. sativa* MsCHS2 (AAA02824.1), *A. thaliana* AtCHS (AAA32771.1), and *P. patens* PpCHS (ABB84527.1) were obtained from the GenBank.

In the FtCHS proteins, a total of fifteen conserved motifs (motifs 1−15) were identified (Figure 5). The number of conserved motifs varied among proteins, ranging from 4 to 10. Proteins belonging to the same phylogenetic group showed comparable motif compositions and arrangements. Notably, motifs 1 and 3−6 were found within the Chal−sti−synt_N domain, whereas motifs 2, 7, and 8 were positioned within the Chal−sti−synt_C domain (Table 3). Apart from FtCHS11, which was missing part of its Chal−sti−synt_N domain, all other FtCHS proteins exhibited well-conserved Chal−sti−synt_N and Chal−sti−synt_C domains (Figure 5). These motifs, particularly those containing the Chal−sti−synt_N and Chal−sti−synt_C domains, suggested their potential significance in CHS protein function. Additionally, three active site residues, Cys164, His303, and Asn336, were, respectively, located in motifs 1, 2, and 7 (Figure 5 and Figure 6 and Table 3). The Cys−His−Asn (CHN) catalytic triad, inherited from ancestral type III polyketide synthases (PKS III) [7,51], remained highly conserved among all FtCHS proteins (Figure 6). Notably, some short motifs (motifs 11−15) were predominantly found in either Group I or Group III (Figure 5), indicating high variability in the N-terminal motifs of FtCHS proteins.

The well-characterized “gatekeeper” phenylalanine residues at positions 215 and 265 of CHS proteins, which are critical for CoA−binding [51], are also conserved. Position 215 was strictly conserved in all FtCHS proteins, while position 265 was highly conserved in most (10/14) FtCHS proteins, with the exception of FtCHS7, FtCHS11, FtCHS12, and FtCHS13 (Figure 6). Notably, FtCHS7 exhibited an amino acid substitution from phenylalanine to threonine (F265T), whereas FtCHS11, FtCHS12, and FtCHS13 displayed a substitution from phenylalanine to leucine (F265L). These substitutions likely resulted in remarkable functional alterations that could influence substrate specificity. Furthermore, the Pro375 residues, which are specific to the CHS family [51], were consistently conserved in all FtCHS proteins (Figure 6). These results suggested strong sequence similarity between FtCHSs and MsCHS2, as well as other plant CHSs, indicating that the CHS family remains evolutionarily conserved.

### 3.6. Analysis of Promoter Cis-Regulatory Elements in FtCHS Genes 

**Figure 7:**
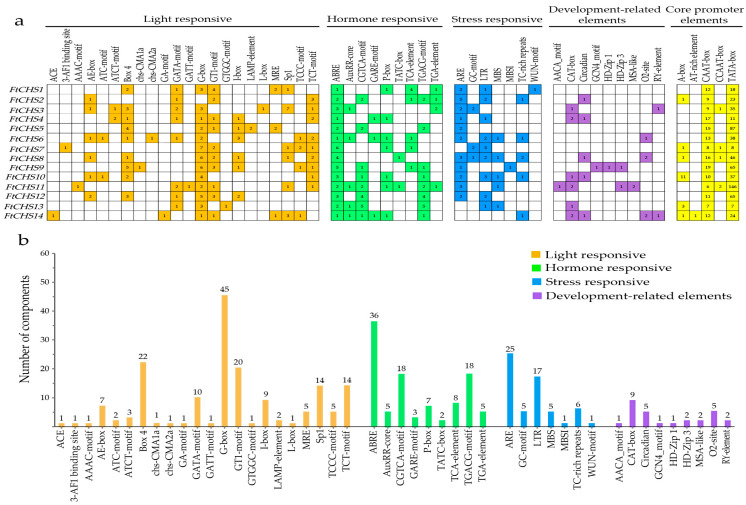
Examination of *cis*−regulatory elements within the promoters of *FtCHS* genes. (**a**) Types and distribution of various *cis*−regulatory elements across each *FtCHS* gene promoter. (**b**) Numbers of different types of *cis*−regulatory elements.

The analysis of the *FtCHS* gene promoters involved evaluating the 2 kb upstream regions from the ATG start codon for each gene. Besides identifying core promoter elements like the TATA−box and CAAT−box, four types of *cis*−regulatory elements were detected: those responsive to light, hormones, stress, and developmental factors (Figure 7). A total of 22 unique light−responsive elements were observed across the *FtCHS* genes, with each gene containing between 8 and 9 different types (Figure 7a). Notably, the G−box element was present in nearly all *FtCHS* genes except *FtCHS2*, acting as a key regulatory factor in plant responses to environmental changes. Previous research indicated that mutations in the G−box led to the loss of UV−B induction responsiveness for the cytochrome c oxidase gene *COX5b*−*2* in *A. thaliana* [52]. Additionally, Box 4 and GT1−motif were frequently identified in 9 and 10 *FtCHS* genes, respectively (Figure 7a). These three elements, G−box, Box 4, and GT1−motif, are the most prevalent light−responsive elements in the *FtCHS* genes (Figure 7a,b). Nine hormone−related elements were also identified, including those associated with abscisic acid (ABA) (ABRE), auxin (AUX) (AuxRR−core and TGA−element), methyl jasmonate (MeJA) (CGTCA−motif and TGACG−motif), gibberellin (GA) (GARE−motif, P−box, and TATC−box), and salicylic acid (SA) (TCA−element) (Figure 7a). The ABRE element was found in all *FtCHS* genes, while seven genes contained both the CGTCA−motif and the TGACG−motif. These three elements (ABRE, CGTCA−motif, and TGACG−motif) are the most abundant hormone−responsive elements in the *FtCHS* genes (Figure 7b), indicating that AUX and MeJA significantly influence the expression of *FtCHS* genes. Moreover, anaerobic conditions activated the ARE element, and low temperatures triggered the LTR element. Both ARE and LTR elements were present in over 60% of *FtCHS* genes (Figure 7a). The fourth category primarily comprised development−related elements such as the CAT−box, Circadian, and O_2_−site, which regulated meristem expression, cell cycle progression, and zein metabolism, respectively. Collectively, these *FtCHS* genes could play a role in light responses, hormone signaling pathways, stress responses, and development−related processes.

### 3.7. Tissue Expression Patterns of FtCHS Genes

**Figure 8:**
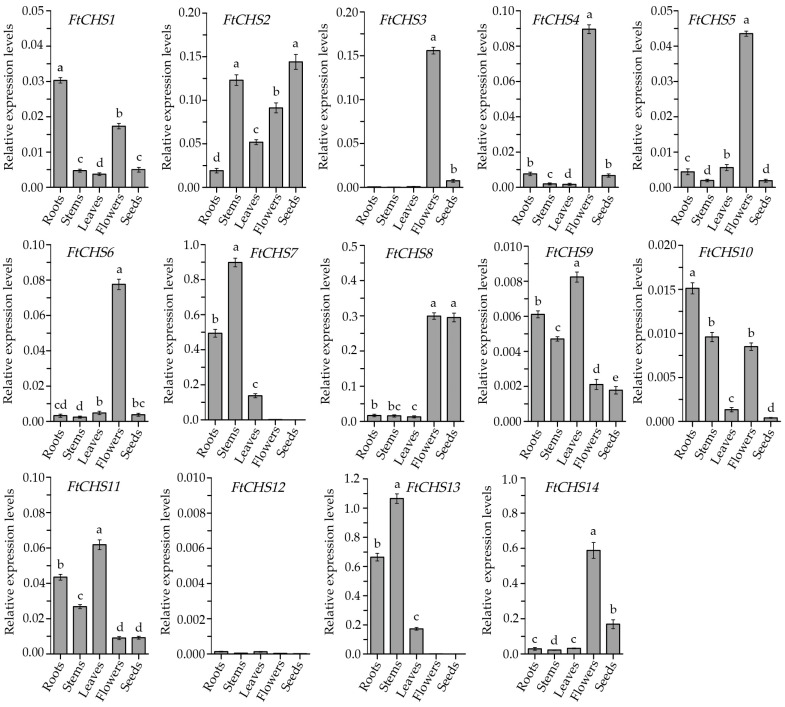
Expression levels of *FtCHS* genes in different tissues. Tissues labeled with different lowercase letters show significant differences (*p* < 0.05) in expression levels.

In this study, qRT−PCR analysis of *FtCHS* gene expression across various tissues of Tartary buckwheat, including roots, stems, leaves, flowers, and seeds, revealed distinct tissue−specific patterns among different *FtCHS* genes (Figure 8). Notably, five *FtCHS* genes, *FtCHS3*, *FtCHS4*, *FtCHS5*, *FtCHS6*, and *FtCHS14*, showed significantly elevated expression levels specifically in floral organs. Likewise, *FtCHS7* and *FtCHS13* exhibited the greatest expression levels in stems. Typically, the expression patterns of genes can provide insight into their specific functions; thus, the observed similarities in expression patterns among these *FtCHS* genes may suggest potential shared biological roles. Additionally, *FtCHS8* demonstrated consistently high expression levels in both flowers and seeds. Conversely, other *FtCHS* genes (*FtCHS1*, *FtCHS2*, *FtCHS9*, *FtCHS10*, and *FtCHS11*) displayed variable expression levels across different tissues. Finally, the expression of *FtCHS12* was nearly undetectable in all examined tissues, indicating its likely non−functional role or limited involvement in the growth and development of Tartary buckwheat.

## 4. Discussion

Members of the *CHS* gene family are recognized for their essential roles in regulating the growth and development of plants [53,54]. The number of *CHS* genes differs markedly among various plant species. For example, *A. thaliana* has a single *CHS* gene [55], whereas *Prunus avium* [54], *Brassica rapa* [10], *C. nankingense* [9], *G. barbadense* [8], *V. corymbosum* [11], and *Triticum aestivum* [56] possess 3, 10, 16, 20, 22, and 87 *CHS* genes, respectively. This diversity underscores the considerable variation in the number of *CHS* genes across different plant species. In Tartary buckwheat, a prior study identified 12 *CHS* genes [34]. Nevertheless, our thorough genome−wide analysis uncovered a total of 14 *CHS* genes in Tartary buckwheat. Among these, nine genes have been previously reported [34], whereas five novel members were identified: *FtCHS1*, *FtCHS7*, *FtCHS10*, *FtCHS11*, and *FtCHS13* (Table 1). Furthermore, an earlier study reported 10 *CHS* genes in Tartary buckwheat [57]. However, only seven of these were found in our analyses. The discrepancy may be attributed to the updated version of the Tartary buckwheat genome annotation file, which has improved annotation accuracy and more precise gene family identification (https://www.mbkbase.org/Pinku1/ (accessed on 8 January 2025)). In large plant genomes, multi−gene families typically arise as a result of whole−genome duplication and domestication processes [58]. The genome of Tartary buckwheat has undergone an independent whole−genome duplication event after its divergence from a common ancestor [34]. Among the 14 *FtCHS* genes identified, one pair of tandemly duplicated genes (*FtCHS3*\*FtCHS4*) was observed, sharing 99.49% similarity in their amino acid sequences. The estimated Ka/Ks ratio for this duplicated *FtCHS* gene pair was found to be below 1.0 (Figure 2), indicating that these genes have experienced purifying selection. This process has led to the removal of deleterious mutations and the preservation of functional stability throughout their evolutionary history [59,60]. Therefore, it can be inferred that the expansion of the *CHS* gene family has likely contributed to the high flavonoid content characteristic of Tartary buckwheat.

Based on molecular evolution analysis, the majority of *CHS* genes can be grouped into two or more subfamilies [54]. In our study, all identified *FtCHS* genes were classified into four primary categories: Groups I, II, III, and IV, according to their phylogenetic relationships (Figure 3). In addition, *FtCHS* members within the same subgroup exhibited highly similar motif distributions and comparable exon−intron structures, suggesting they share similar fundamental functions (Figure 4 and Figure 5). Conserved motifs 1 and 3−6 were identified within the Chal−sti−synt_N domain, whereas motifs 2, 7, and 8 were localized in the Chal−sti−synt_C domain (Table 3). These two domains encompass a buried active site comprising several amino acid residues, including Cys164, Phe215, His303, and Asn336, which constitute the catalytic machinery [43,51]. The presence of these two structural domains across all FtCHS proteins suggested their conserved role in catalyzing chalcone compound formation (Table 3, Figure 5). Moreover, the catalytic triad composed of Cys−His−Asn (CHN), situated within motifs 1, 2, and 7, plays an essential role in catalytic activity and was identified in all FtCHS proteins (Table 3). This suggests that this catalytic triad, which has been inherited from the PKS III ancestor [51], exhibits high conservation. All FtCHS proteins contained the CHS family−specific Pro375 and CoA−binding residues (Phe215 and Phe265) (Figure 6), further supporting their conserved evolution, although some substitutions at position Phe265 probably resulted in different substrate preferences. Importantly, multiple sequence alignment of the identified FtCHS proteins with MsCHS2, AtCHS, and PpCHS revealed high similarity, suggesting conservation of the CHS family proteins across different plant species. Additionally, the analysis of gene structure and conserved motif corroborated the phylogenetic analysis and supported the hypothesis that *FtCHS* genes have remained highly conserved throughout evolution. Notably, most *FtCHS* genes (9/14) contained two exons and one intron (Figure 4), which is consistent with the previously proposed structure that is composed of two exons and one intron in other plants [8,9,10].

Gene expression patterns serve as a critical reflection of gene function, with genes sharing common evolutionary origins or regulated by the same transcription factors often exhibiting similar expression profiles [61,62]. Considering the varied roles of *CHS* genes, their expression levels can change in response to particular environmental conditions or may be specific to certain organs or developmental stages. In this investigation, we examined the expression levels of all 14 *FtCHS* genes across different tissues (Figure 8). Notably, several *FtCHS* genes exhibited variable expression patterns depending on the tissue type. For example, *FtCHS1*, *FtCHS2*, *FtCHS9*, *FtCHS10*, and *FtCHS11* showed distinct expression levels across various tissues. Conversely, some *FtCHS* genes displayed highly similar expression patterns. Specifically, *FtCHS3*, *FtCHS4*, *FtCHS5*, and *FtCHS6* were highly and specifically expressed in Tartary buckwheat flower organs (Figure 8). Furthermore, these four genes were clustered within the same subgroup (Group II) in the phylogenetic analysis (Figure 3), suggesting they may play analogous roles in the same biochemical pathway related to flowers. Previous studies have shown that rutin accumulation in Tartary buckwheat flowers is significantly higher compared to roots, stems, leaves and seeds [33,34]. Therefore, it is plausible that the specific expression of these four *FtCHS* genes in Tartary buckwheat flowers is associated with rutin synthesis and accumulation. Additional functional validation is required for these important *FtCHS* genes, as they are potentially implicated in the flavonoid synthesis pathway within Tartary buckwheat.

## 5. Conclusions

In this study, we identified and analyzed the fundamental characteristics of 14 *FtCHS* genes (*FtCHS1*−*FtCHS14*) from tartary buckwheat to explore their potential functions. Our analysis of gene architectures, conserved motifs, evolutionary patterns, and tissue−specific expression profiles revealed that the *CHS* gene family in tartary buckwheat has remained relatively conserved during evolution. By integrating tissue−specific expression data with phylogenetic relationships, we proposed that four *FtCHS* genes, *FtCHS3*, *FtCHS4*, *FtCHS5*, and *FtCHS6*, are likely to play analogous biological roles in tartary buckwheat flowers. This research enhances our understanding of *FtCHS* genes and contributes to the exploration of their molecular evolution, expression, and regulation. Moreover, it provides promising candidates for future functional studies.

## Figures and Tables

**Table 1:** Identification of the *CHS* gene family members in Tartary buckwheat.

Gene Name	Gene ID	Chromosomal Position	Open Reading Frame (bp)	Coding Sequence (bp)	Number of Exons
*FtCHS1*	FtPinG0000291700.01	Chr1: 4,675,628–4,676,999 (+)	1179	1176	2
*FtCHS2*	FtPinG0008131000.01	Chr2: 10,868,328–10,869,961 (−)	1182	1179	3
*FtCHS3*	FtPinG0003701300.01	Chr2: 53,644,013–53,645,287 (+)	1173	1170	2
*FtCHS4*	FtPinG0003701500.01	Chr2: 53,650,904–53,652,193 (+)	1173	1170	2
*FtCHS5*	FtPinG0003710800.01	Chr2: 53,836,790–53,838,140 (−)	1173	1170	2
*FtCHS6*	FtPinG0006832300.01	Chr4: 15,131,108–15,132,422 (−)	1176	1173	2
*FtCHS7*	FtPinG0008741700.01	Chr4: 53,611,525–53,613,020 (+)	1155	1152	3
*FtCHS8*	FtPinG0000551600.01	Chr5: 1,802,553–1,804,267 (−)	1188	1185	2
*FtCHS9*	FtPinG0001351300.01	Chr5: 11,342,805–11,353,551 (+)	1161	1158	8
*FtCHS10*	FtPinG0002783200.01	Chr7: 2,444,081–2,445,384 (−)	1158	1155	2
*FtCHS11*	FtPinG0009022800.01	Chr7: 7,164,510–7,169,577 (−)	1035	1032	4
*FtCHS12*	FtPinG0002110600.01	Chr7: 9,248,374–9,257,781 (+)	1158	1155	4
*FtCHS13*	FtPinG0002106500.01	Chr7: 9,413,803–9,416,602 (−)	1155	1152	4
*FtCHS14*	FtPinG0008806400.01	Chr7: 32,140,231–32,141,882 (−)	1182	1179	2

**Table 2:** The physicochemical properties of the FtCHS proteins.

Protein Name	Protein Length (aa)	Molecular Weight (Da)	Isoelectric Point	Instability Index	Grand Average of Hydropathicity	Subcellular Localization
FtCHS1	392	42,274.70	5.98	37.16	−0.083	Cytoplasm
FtCHS2	393	43,177.87	5.85	40.75	−0.113	Cytoplasm
FtCHS3	390	43,646.41	6.18	37.00	−0.215	Cytoplasm
FtCHS4	390	43,621.40	6.24	37.17	−0.212	Cytoplasm
FtCHS5	390	43,661.46	6.53	37.50	−0.223	Cytoplasm
FtCHS6	391	43,728.64	7.58	36.17	−0.219	Cytoplasm
FtCHS7	384	41,933.31	5.90	36.78	0.013	Cytoplasm
FtCHS8	395	43,438.97	5.67	37.90	−0.156	Cytoplasm
FtCHS9	386	42,299.78	5.66	35.82	−0.013	Cytoplasm
FtCHS10	385	42,210.40	5.38	36.97	−0.167	Cytoplasm
FtCHS11	344	37,702.24	5.77	40.59	−0.144	Cytoplasm
FtCHS12	385	42,398.83	6.14	46.42	−0.052	Cytoplasm
FtCHS13	384	41,904.86	5.61	33.25	−0.065	Cytoplasm
FtCHS14	393	43,208.72	5.78	36.52	−0.150	Cytoplasm

**Table 3:** Detailed information of the 15 conserved motifs in FtCHS proteins.

Motif	Width (aa)	Best Possible Match Sequence	Domain
1	119	SLDARQEMAITEVPKLGKEAAZKAIKEWGQPKSKITHLIMCTTSGVDMPGADYQLAKLLGLRPSVKRFMVYQQG**C**FAGGTVLRLAKDJAENNKGARVLVVCSEITAIGFRGPSETHJDS	Chal−sti−synt_N
2	159	LSIEKPIFELVWTSQTIJPDSEGAIEGHLREAGLTFHLTKDVPQLISNNIEKCLLEAFSPLNITDWNSIFWIA**H**PGGPAILDQIEEKLGLKKEKLRATRQVLNDYG**N**MSSACVLFVMDEMRKKSLENGHATTGEGLDWGVLFGFGPGLTVETVVLRSVP	Chal−sti−synt_C
3	80	QZIRKAQKANGPATVLAIGTATPPTCYPQSEYPDFYFRVTKSEHMTELKQKFKRICDKSGIEKRFMFLTEEILTPNPNMC	Chal−sti−synt_N
4	21	LVGQALFGDGAGAVIVGABPB	Chal−sti−synt_N
5	39	QFKEKFKRICEKSMIEKRYTHLTEEILKENPNIAGYDSP	Chal−sti−synt_N
6	35	GAATILAIGTANPPQCIYQADFPDGYFRATNSDHL	Chal−sti−synt_N
7	111	QQFLPGTDGIIDGGLTEEGINFKLGRELPQKIEENIEGFCGKJLGKIGDEKEGYNDLFWAV**H**PGGPAILNRJEKKLGLEGEKLEASRKALADFG**N**VSSNTIFYVMEYMREE	Chal−sti−synt_C
8	20	EWGLGLAFGPGITFEGILIR	Chal−sti−synt_C
9	6	TTTLAN	*
10	7	KPFFELN	*
11	11	KKNEDGSREQC	*
12	6	MAKDVD	*
13	6	MAATLD	*
14	6	PNGRTP	*
15	6	KKEEIE	*

The asterisk (*) signifies the lack of motifs within the domain sequences.

## Data Availability

The genome data utilized in this study were obtained from the Tartary buckwheat database (https://www.mbkbase.org/Pinku1/ (accessed on 8 January 2025)).

## Supplementary Materials

The following supporting information can be downloaded at https://www.mdpi.com/article/10.3390/genes16040451/s1, Table S1: CHS proteins from different plants for phylogenetic analysis; Table S2: Specific primers of *FtCHS* genes for qRT−PCR analysis.
